# Discrimination of Shellac and Nitrocellulose Varnishes Using Ambient Mass Spectrometry and Partial Least Squares Regression

**DOI:** 10.3390/molecules31203772

**Published:** 2026-10-20

**Authors:** Štěpán Dostál, Vilém Urban, Lukáš Kučera

**Affiliations:** 1 Department of Analytical Chemistry, Faculty of Science, Palacký University, 17. Listopadu 12, 77900 Olomouc, Czech Republic; stepan.dostal@upol.cz; 2 Department of Art History, Faculty of Arts, Palacký University, Univerzitní 3, 77900 Olomouc, Czech Republic; vilemurban@seznam.cz

**Keywords:** lacquers, mass spectrometry, infrared spectroscopy, cultural heritage

## Abstract

Historical surface coatings on Czechoslovak furniture from the interwar period (1918–1945) frequently combine shellac and nitrocellulose lacquers, yet their compositional characterisation in aged specimens remains analytically challenging. We report the first application of atmospheric solids analysis probe mass spectrometry (ASAP-MS) combined with partial least squares regression for semiquantitative estimation of shellac/nitrocellulose ratios in historical varnish systems. ATR-FTIR spectroscopy combined with principal component analysis represents a standard tool in conservation practice for varnish characterisation; however, our comparative evaluation demonstrated that this approach is limited to qualitative discrimination and does not support reliable semiquantitative composition estimates. The ASAP-MS workflow was applied to microsamples from an authenticated historical furniture object of Czech origin (ca. 1939), yielding direct compositional estimates that can guide the selection of appropriate restoration treatments and reduce the risk of chemically incompatible interventions.

## 1. Introduction

Surface finishes represent a key component of historical furniture, as they directly determine both the visual appearance and the material integrity of objects. In the case of Czechoslovak furniture production between 1918 and 1945, this aspect is of particular importance due to a rapid technological transition in coating materials. Alongside traditional natural substances such as shellac, industrial practice increasingly adopted early synthetic polymers, most notably nitrocellulose. These materials were widely applied in mass-produced furniture, often in the form of multilayer or mixed varnish systems combining natural and synthetic components.

Despite the technological and historical significance of this transition, the material composition of such surface finishes remains insufficiently understood. While art historical and design-oriented research has extensively addressed Czechoslovak furniture of the early 20th century, the chemical characterization of its surface treatments has received comparatively little attention. In particular, varnish systems based on combinations of shellac and nitrocellulose have not been systematically studied, and their occurrence, composition, and variability within historical objects remain only partially documented.

Shellac and nitrocellulose, frequently encountered in Czechoslovak furniture finishes from the first half of the twentieth century, differ substantially in their chemistry and ageing behaviour. Shellac is a natural resin composed mainly of esters and polyesters of hydroxyaliphatic and sesquiterpenoid acids; ageing-related self-esterification and crosslinking can reduce its alcohol solubility and increase brittleness [1]. Nitrocellulose lacquers contain cellulose nitrate, typically combined with plasticizers and modifying resins; ageing can involve denitration and carbonyl formation, altering coating properties and removability [2]. In mixed finishes, the relative proportions of these materials influence coating properties and their response to conservation treatments. Qualitative identification alone therefore provides an incomplete basis for restoration decisions, motivating semiquantitative assessment of their relative contributions. The situation is further complicated by the compositional complexity of varnish systems. Shellac and nitrocellulose, which are frequently encountered in Czechoslovak furniture finishes of this period, differ substantially in their chemical nature and physical behavior. When present in mixtures, their relative proportions influence key properties of the coating, including its optical appearance, mechanical stability, and ageing processes. From a conservation perspective, simple identification of these materials is therefore insufficient; a more detailed understanding of their relative contribution within the varnish layer is required.

Consequently, there is a need for analytical approaches capable of providing deeper insight into the composition of such historically relevant, yet insufficiently characterized, varnish systems. Addressing this problem is essential for improving the reliability of conservation practice and for ensuring that restoration interventions remain compatible with the original materials used in Czechoslovak furniture production of the early 20th century.

Against this conservation background, the analytical investigation of historical varnishes typically relies on a combination of complementary techniques. Optical and vibrational spectroscopic methods are commonly employed as first-line screening tools, owing to their non-destructive character, minimal sample requirements, and suitability for in situ or micro-invasive analysis [3,4]. Among vibrational methods, Fourier transform infrared spectroscopy (FTIR), including attenuated total reflectance (ATR-FTIR) and micro-FTIR configurations, is widely used for rapid screening of coating materials and for assessing their stratigraphy and composition [5,6]. For nitrocellulose-based lacquers, FTIR is particularly valuable because the cellulose nitrate component exhibits characteristic nitrate bands, which have been used to identify such coatings on historical furniture and other heritage objects [2,7]. Raman spectroscopy can provide complementary non-destructive molecular information for the study of organic binders and varnishes in heritage objects [8]. However, spectroscopic approaches also show important limitations when applied to aged and chemically complex historical varnishes. Their diagnostic power may decrease as sample composition becomes more heterogeneous or altered by degradation, meaning that the results are often best interpreted in combination with other analytical methods. In the case of Raman spectroscopy, fluorescence from organic materials may represent an additional practical limitation [9,10,11].

For more specific molecular characterization, varnish analysis commonly employs chromatography–mass spectrometry approaches, particularly gas chromatography–mass spectrometry (GC–MS), including pyrolytic variants (Py-GC/MS), and liquid chromatography–mass spectrometry (LC–MS). Py-GC/MS requires minimal sample preparation and yields diagnostic markers even from polymerized or poorly soluble materials. Extraction-based GC–MS, which typically requires derivatization, and LC–MS offer higher molecular specificity at the cost of more demanding sample preparation and destructive microsampling [12,13,14]. Both GC–MS (including pyr-GC/MS) and LC–MS approaches have been successfully applied to shellac resins, supporting the characterization of their molecular composition, oligomeric fractions, and degradation products [15,16,17,18]. Additional MS-based approaches such as time-of-flight secondary ion mass spectrometry (ToF-SIMS) can provide spatially resolved chemical information from varnishes [19]. For varnish analyses, a more recent approach utilizes ambient techniques such as atmospheric solids analysis probe mass spectrometry (ASAP-MS). ASAP-MS is a direct ambient ionization technique in which analytes deposited on a glass capillary are thermally desorbed in a heated nitrogen stream and ionized via an APCI-like mechanism, enabling rapid analysis of solid and liquid samples with minimal sample preparation and without prior chromatographic separation [20]. Ambient techniques principal limitation is that the recorded spectra are composite fingerprints susceptible to signal overlap, matrix effects, and thermal or in-source fragmentation, making chemometric evaluation essential for robust interpretation [21]. In the context of historical lacquer analysis, ASAP-based fingerprinting in combination with exploratory principal component analysis (PCA) has been so far demonstrated in a study on Asian lacquers, where it enabled discrimination of samples of different geographic origin [22]. Overall, ASAP-MS can be regarded as a promising emerging tool for lacquer analysis, although its application has so far been documented, to date, only in a single qualitative study by Zemek et al. [22].

## 2. Research Aim

The present study explores a rapid ambient MS workflow for the analysis of historically relevant mixed varnish systems based on shellac and nitrocellulose. Using atmospheric solids analysis probe mass spectrometry (ASAP-MS) combined with partial least squares regression (PLS-R), we developed a semiquantitative model to estimate the relative proportions of both components in model mixtures. As ATR-FTIR remains one of the most widely used analytical techniques in cultural heritage practice, the same sample set was also analyzed by ATR-FTIR to provide an established point of reference. This complementary comparison was included to place the proposed ASAP-based approach into the context of current conservation analysis.

## 3. Results and Discussion

### 3.1. ATR-FTIR

**Figure 1:**
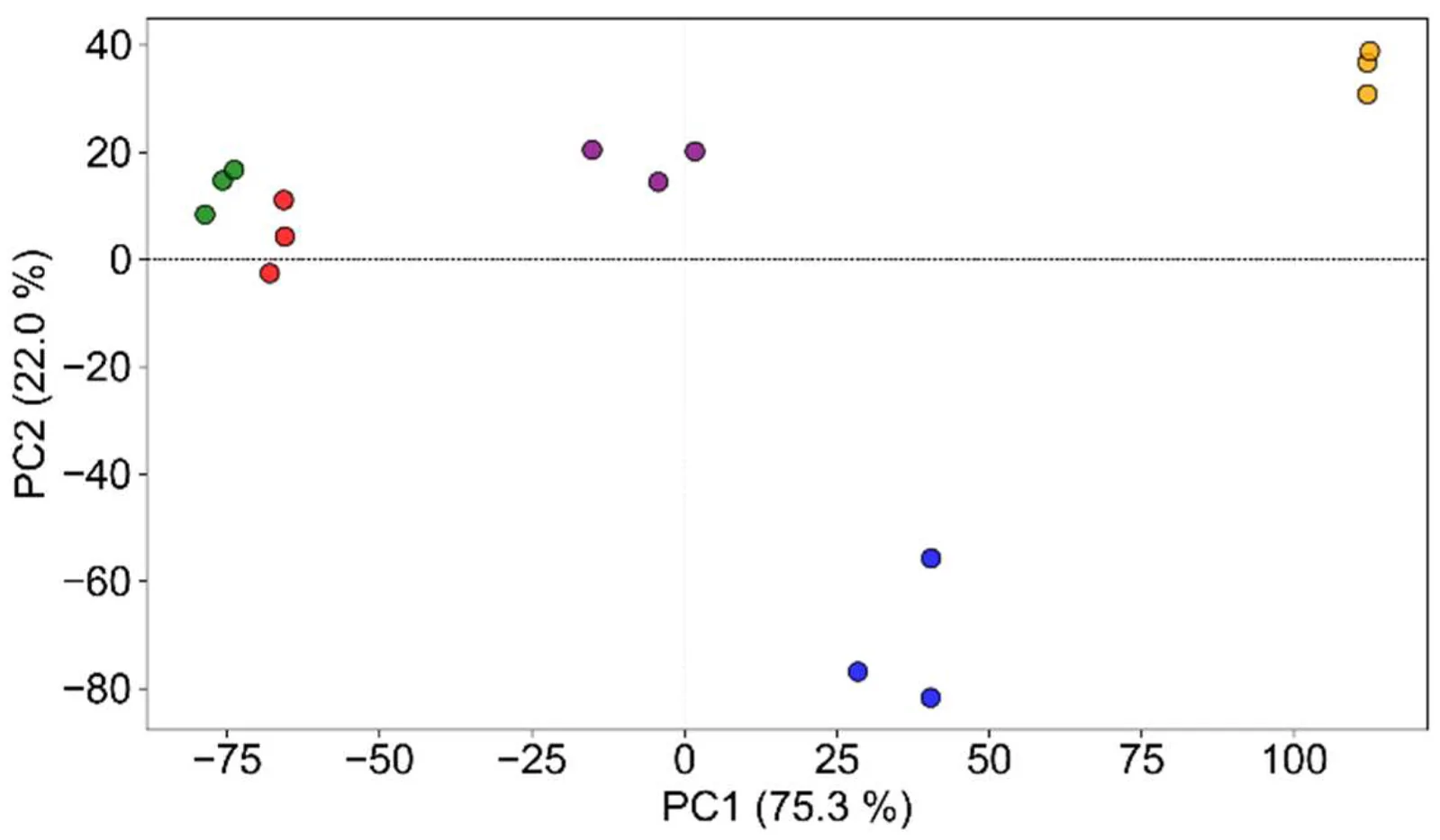
PCA plot (PC1 vs. PC2; 75.3% and 22.0% of explained variance, respectively) obtained by FTIR analysis representing group separation of pure varnishes and their binary mixtures. (Nitrocellulose (blue), pure shellac (orange), and model mixtures S/NC 50/50 (green), 25/75 (red), and 75/25 (purple)).

ATR-FTIR/PCA was included as an established qualitative reference approach in conservation practice, enabling comparison with the semiquantitative information obtained independently by ASAP-MS/PLS-R. Unit-variance scaling was applied prior to analysis. Each sample group was measured in triplicate. As shown in Figure 1, the PCA score plot reveals clear separation of all five sample groups along the first two principal components, which together account for 97.3% of the total spectral variance (PC1: 75.3%, PC2: 22.0%). Pure shellac and pure nitrocellulose are located in opposite regions of the score plot, reflecting their substantially different chemical composition. The three binary mixtures occupy intermediate positions between the pure component clusters. Replicates within each group form tight clusters, demonstrating good measurement repeatability. These results indicate that ATR-FTIR combined with PCA can allow qualitative discrimination of shellac, nitrocellulose, and their binary mixtures at the tested mass ratios.

### 3.2. ASAP-MS

Because ASAP sampling is performed by loading material onto a glass capillary and thermally desorbing it in a heated nitrogen stream, the exact amount of sample introduced into the source cannot be controlled as rigorously as in a conventional volumetric injection. In addition, the overall signal may vary during acquisition as the deposited material is progressively depleted. For this reason, the spectra were pretreated by TIC normalization and log_10_ transformation (Section 4.4), which reduced spectrum-to-spectrum differences in total ion signal, improved comparability across samples, and compressed the dynamic range prior to chemometric analysis.

To improve ion recovery across compounds of different volatility, data acquisition was performed using a pseudo temperature program (the starting desorption temperature was 200 °C and was increased to 500 °C after 10 s) rather than a single fixed desolvation temperature. In ASAP-MS, temperature gradients promote more gradual desorption according to volatility/boiling behavior and can provide broader and more informative spectral coverage than constant-temperature operation, whereas excessively high fixed temperatures may alter the spectra through increased thermal fragmentation or pyrolysis. In our optimization experiments, the 200–500 °C ramp therefore provided the most suitable data quality, while fixed temperatures of 500 °C and 600 °C were not retained for subsequent measurements.

#### 3.2.1. Principal Component Analysis

**Figure 2:**
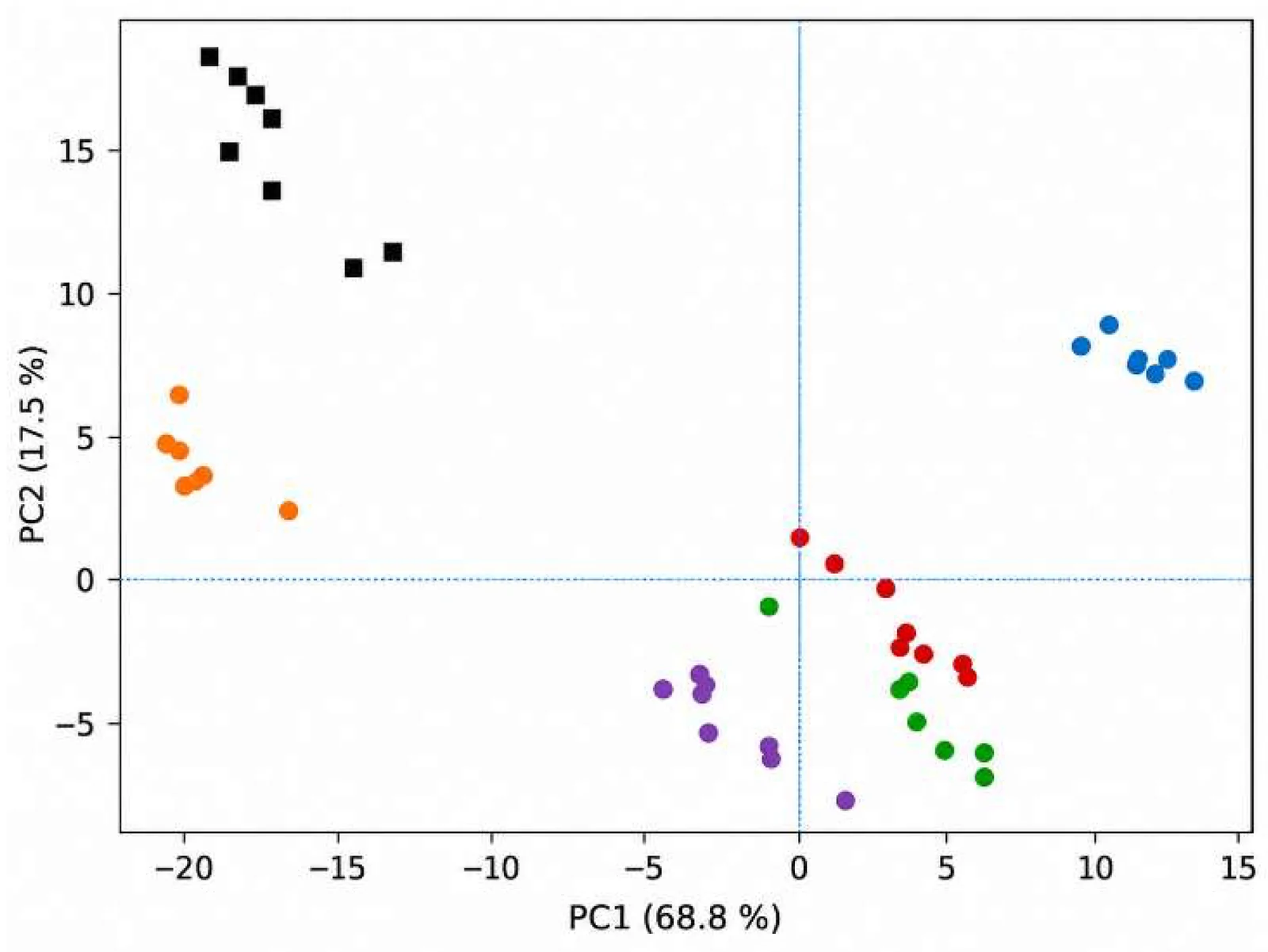
PCA score plot (PC1 vs. PC2; 68.8% and 17.5% of explained variance, respectively) of model samples analysed by ASAP-MS. Blank samples (black squares) are shown together with pure nitrocellulose (blue), pure shellac (orange), and model mixtures S/NC 50/50 (green), 25/75 (red), and 75/25 (purple).

Processed spectra (Section 4.4) of model samples of shellac and nitrocellulose varnishes and their mixtures were first evaluated by PCA as an exploratory quality-control tool to verify replicate consistency, identify potential outliers, and assess overall sample grouping; the main semiquantitative evaluation was then performed using PLS-R. As shown in Figure 2, clear separation of the groups was observed. Samples formed six distinct clusters corresponding to blank, shellac, nitrocellulose, and their mixtures with mass ratios of 75/25, 50/50, and 25/75. Blank samples were located in the upper left part of the score plot, clearly separated from all varnish-containing samples. Pure shellac and pure nitrocellulose samples were also separated from each other, while the mixture groups were positioned between them according to their composition. In particular, the 75/25 shellac/nitrocellulose mixture was located closer to the shellac group, the 25/75 mixture was closer to the nitrocellulose group, and the 50/50 mixture remained between these two extremes. PC1 and PC2 together explained more than 86% of the total variance in the dataset. Overall, the PCA results confirmed good data consistency and composition-dependent sample grouping, thus supporting the subsequent use of PLS-R as the principal semiquantitative approach.

#### 3.2.2. Partial Least Square Regression

**Figure 3:**
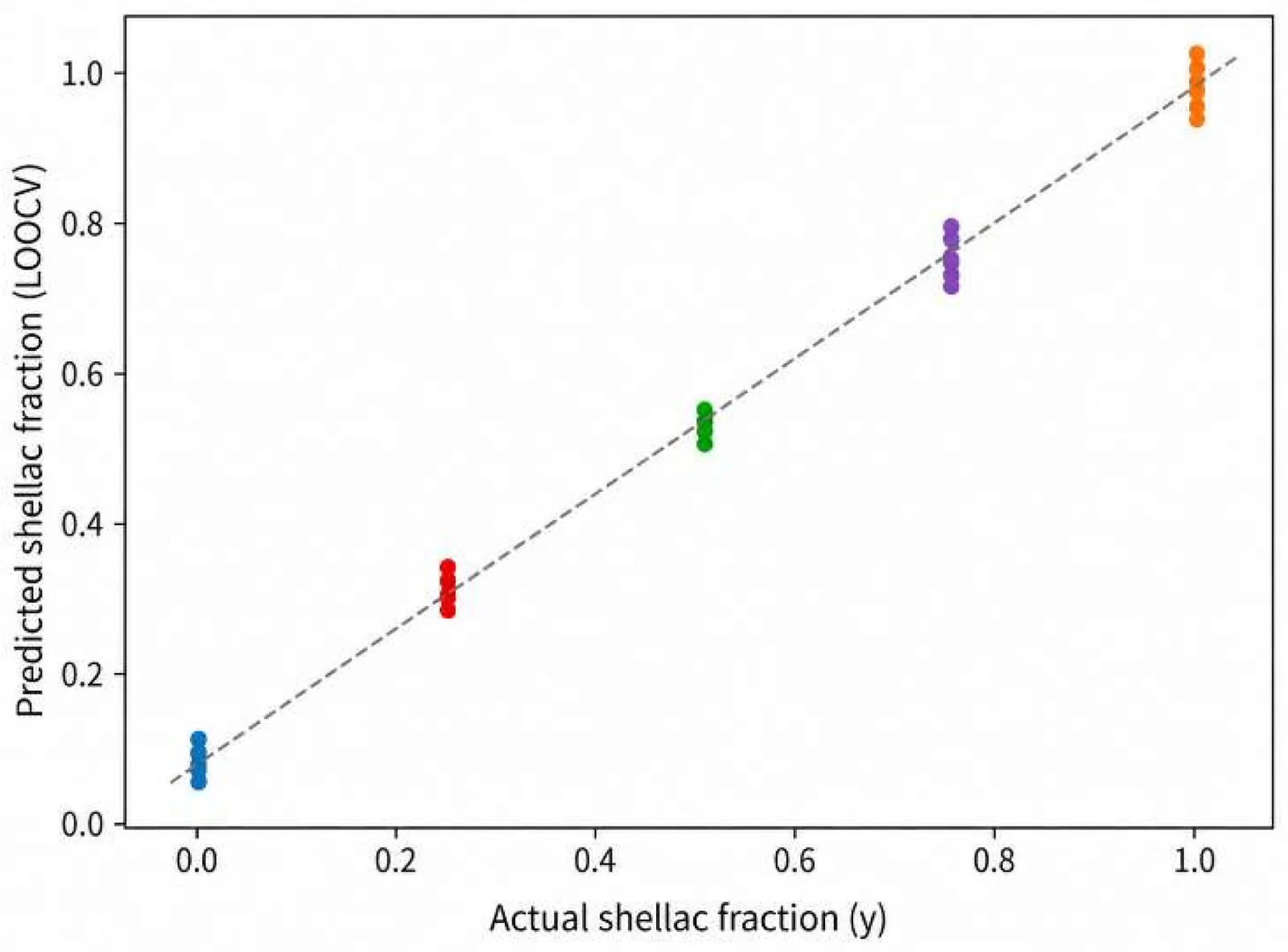
Leave-one-out cross-validated PLS-R prediction of shellac fraction in model shellac/nitrocellulose mixtures (Q^2^ = 0.995, RMSE = 0.024). Predicted versus actual shellac mass fraction: pure nitrocellulose (NC, blue; y = 0.00), Š/NC 25/75 (V2, red; y = 0.25), Š/NC 50/50 (V1, green; y = 0.50), Š/NC 75/25 (V3, purple; y = 0.75), and pure shellac (S, orange; y = 1.00).

Under the final optimized conditions, the PLS-R model showed very strong predictive performance (Figure 3). The 0.02 Da binning strategy combined with the final preprocessing workflow yielded a cross-validated coefficient of determination of approximately 0.995, a root mean square error of cross-validation of about 0.024, a slope close to 0.993, and an intercept close to zero (0.0021). These values indicate that the predicted shellac fractions closely followed the reference composition values over the full calibration range. The high cross-validated coefficient of determination demonstrates that the model captured most of the composition-dependent variance in the processed spectra, whereas the low root mean square error of cross-validation indicates that the prediction error remained small relative to the full 0–1 shellac-fraction scale. The near-unity slope and low intercept further suggest the absence of substantial proportional or constant bias in the final model. Taken together, these parameters support the conclusion that the ASAP mass spectrometry fingerprints contained sufficient systematic compositional information to enable reliable semiquantitative modelling of shellac content.

Additional model verification was provided by permutation testing, in which the response vector was randomly permuted five hundred times while the predictor matrix remained unchanged. The final model gave an observed cross-validated coefficient of determination of 0.995, whereas the corresponding permuted models produced markedly lower values, with a median of −0.3252 and a maximum of 0.1367. None of the permuted models approached the predictive performance of the original model, confirming that the final partial least squares regression model reflects genuine composition-related variation in the mass spectral data rather than an incidental statistical association.

At the level of individual calibration groups, the predicted shellac fractions remained very close to the nominal reference values across the full composition range, from pure nitrocellulose to pure shellac. The mean predicted shellac fractions were 0.0002, 0.2564, 0.4953, 0.7430, and 0.9987 for the reference levels 0.00, 0.25, 0.50, 0.75, and 1.00, respectively, confirming the correct compositional ordering and only minimal systematic deviation.

**Figure 4:**
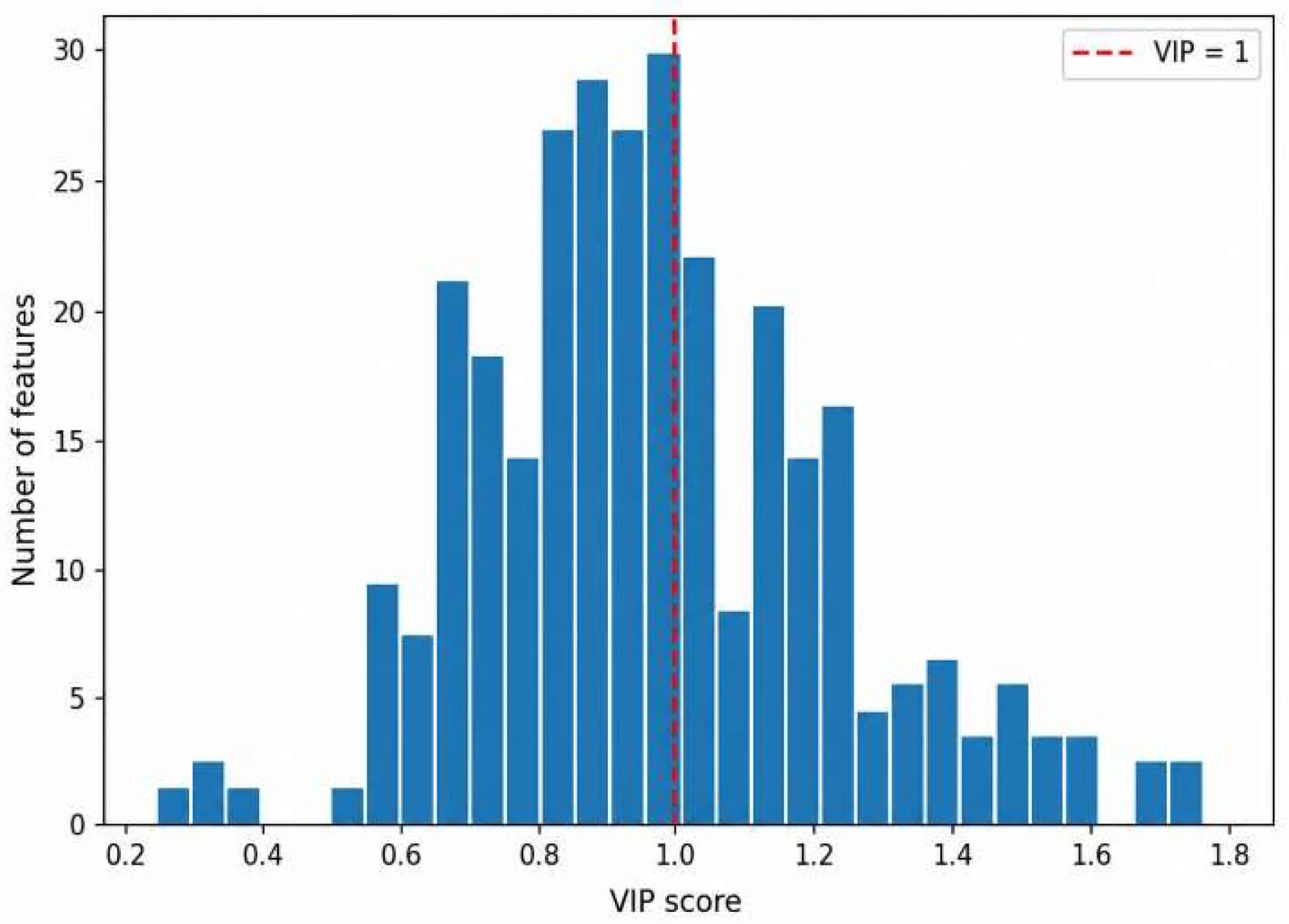
Distribution of variable importance in projection (VIP) scores for the 300 variables included in the PLS-R model.

Feature importance was assessed by computing Variable Importance in Projection (VIP) scores and PLS regression coefficients for the 300 features selected in the final model. All top-50 features ranked by VIP exceeded the threshold of 1.0 (Figure 4), confirming that the pre-selected feature set is dominated by genuinely informative variables rather than noise. The two highest-ranking features (*m*/*z* ≈ 726.3 and *m*/*z* ≈ 632.2, VIP ≈ 1.78) showed a marked separation from the remainder of the ranked list, followed by a gradual decline without a distinct elbow, indicating that the model relies on a broad combination of signals rather than a small number of dominant ions. In total, 114 of the 300 selected features had VIP ≥ 1.0, with the distribution centred near VIP ≈ 1.0 and a pronounced right tail extending to VIP ≈ 1.8.

Inspection of the VIP-versus-regression-coefficient plot revealed a clear directional asymmetry: the majority of high-VIP features (VIP > 1.0) carried positive regression coefficients, identifying them as shellac-associated markers whose abundance increases proportionally with shellac fraction. A smaller subset of high-VIP features exhibited negative coefficients, representing nitrocellulose-associated signals that decrease with increasing shellac content. This directional asymmetry reflects the inherent chemical properties of the two materials rather than a limitation of the model: shellac produces a richer ASAP-MS fingerprint owing to its complex mixture of polyhydroxyaliphatic acids and triterpenoid constituents, whereas nitrocellulose yields a more limited set of diagnostic ions under ASAP conditions [18]. Importantly, nitrocellulose-associated markers with VIP > 1.0 were nonetheless identified, confirming that the model captures discriminative information from both components of the binary system.

### 3.3. Real Sample Analysis and FTIR Comparison

**Figure 5:**
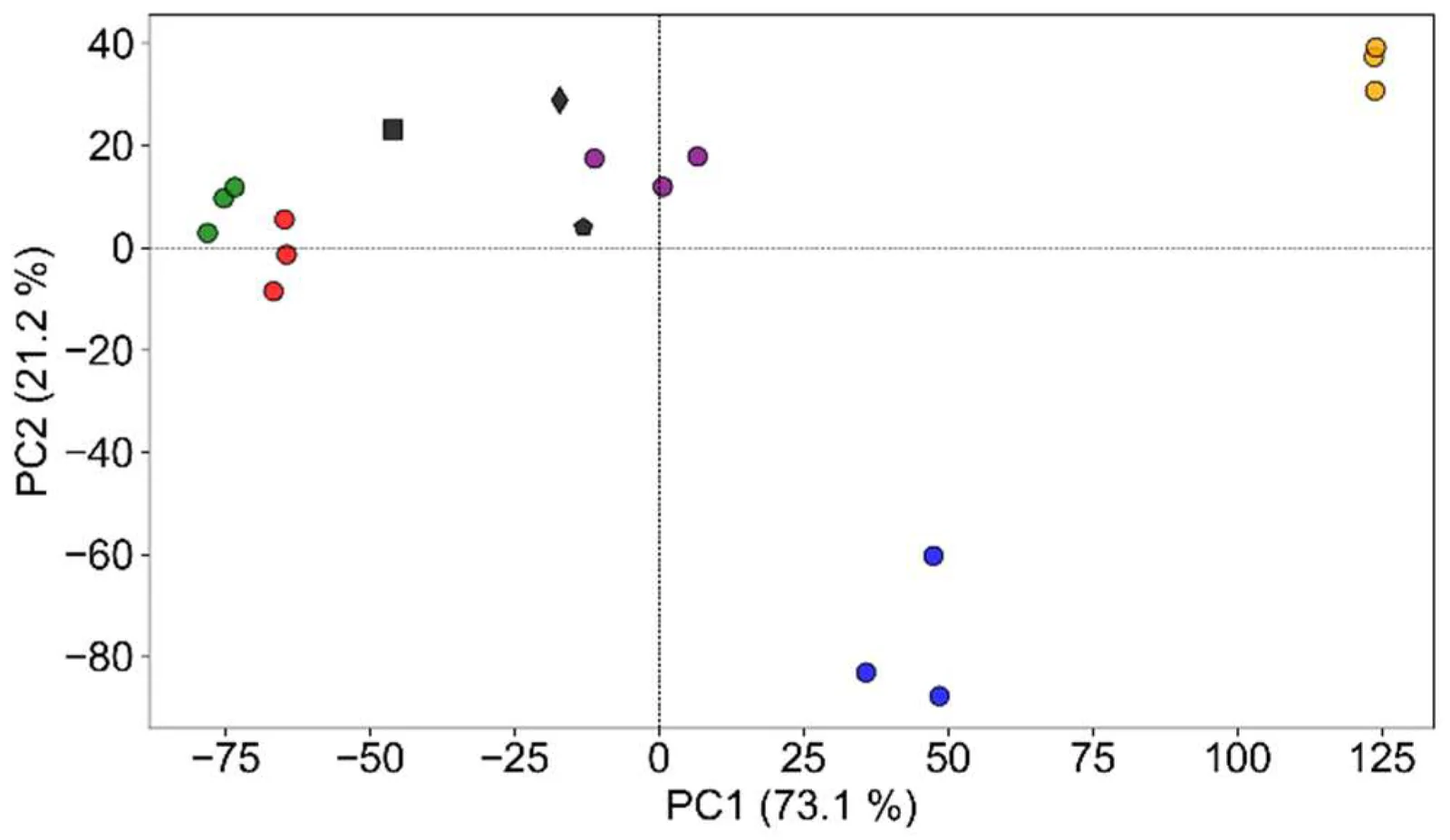
PCA plot (PC1 vs. PC2; 73.1% and 21.2% of explained variance, respectively) from FTIR analysis of model samples with real sample projection. (Nitrocellulose (blue), pure shellac (orange), and model mixtures S/NC 50/50 (green), 25/75 (red), and 75/25 (purple), SKR-A (black square), DVIR-B (black diamond), DESKA-A (black pentagon)).

The three real-object samples—SKR-A, DVIR-B, and DESKA A—were projected onto the PCA score plot derived from the ATR-FTIR spectra (Figure 5). SKR-A projected into the negative PC1 region, in the vicinity of the S/NC 50/50 and S/NC 25/75 reference mixture clusters. However, its score plot position fell in the region where the reference clusters overlap, preventing any more precise compositional inference from the FTIR PCA alone.

DVIR-B projected into the central-left region of the score plot at moderately positive PC2 values, while DESKA A was located near the plot origin. Both samples were assigned to the shellac-dominated class by PCA-based classification, with their score positions falling in close proximity to the S/NC 75/25 reference cluster. This suggests that both samples contain a shellac-dominated composition, though the FTIR PCA does not allow quantitative estimation of the exact shellac-to-nitrocellulose ratio.

This case illustrates a fundamental constraint of PCA-based evaluation: PCA is an exploratory, dimensionality-reduction technique and does not constitute a quantitative or semiquantitative model. Its output—positions in a score space—allows qualitative class comparison but cannot be translated into numerical composition estimates. When a real sample projects near the boundary of multiple reference clusters, as observed here for SKR-A, the approach provides no objective basis for preferring one compositional assignment over another.

After FTIR analysis, the same real-object samples were evaluated by ASAP-MS. The PLS-R model predicted shellac mass fractions for all three samples based on duplicate measurements (Table 1). SKR-A yielded the highest predicted shellac fraction (0.7807 ± 0.004), indicating a shellac-dominated composition. DVIR-B was predicted at a shellac fraction of 0.6392 ± 0.038, also consistent with shellac dominance, though the markedly wider standard deviation relative to the other two samples suggests greater measurement variability for this specimen. DESKA A returned a predicted shellac fraction of 0.3959 ± 0.002, placing it near the equimolar composition boundary and precluding a definitive class assignment on the basis of the PLS-R prediction alone.

As the PLS-R model was calibrated exclusively on binary shellac/nitrocellulose mixtures, the predicted shellac mass fraction implicitly defines the nitrocellulose content as the complementary remainder, under the assumption that no other major components contribute to the spectral signal.

Several caveats apply to both approaches. The calibration samples, though prepared as binary model mixtures, were stored under uncontrolled ambient conditions for approximately two years prior to analysis; this exposure may have introduced oxidative and hydrolytic changes broadly comparable in character, if not in extent, to those occurring in historical specimens. The matrix transfer problem—the application of a model trained on compositionally defined mixtures to genuine historical varnishes of unknown provenance and ageing history—therefore affects both the FTIR PCA projections and the PLS-R predictions, and its magnitude cannot be quantified without reference samples of authenticated composition. Additionally, both models assume a binary shellac/nitrocellulose system; contributions from other components present in real varnish layers would not be accounted for and could distort the compositional inference in either direction. The predicted shellac fractions for the real-object samples should therefore be regarded as provisional semiquantitative estimates, subject to confirmation by an orthogonal method.

## 4. Material and Methods

### 4.1. Studied Materials and Objects

The three real-object samples used in this study, labeled DVÍŘ-B, SKR-A, and DESKA-A, are surface coating samples taken from an authentic historical object. This object is a veneered storage piece of furniture acquired through a donation call for long-term research purposes within the project. To ensure the long-term preservation of this object, it was added to the collection of the Moravian Gallery in Brno (Czech Republic) as an acquisition. The object bears no factory identification label that would unambiguously attribute it to the production and provenance of a specific factory. However, on parts of the structure not normally visible, the item bears factory inscriptions of an instructional nature in the Czech language, as well as a factory inscription on the back panel reading R244 3/5 39. It can be safely assumed that this is a piece of furniture of Czech origin dating from around 1939. From the perspective of furniture typology, this is one of the most common models, widely used as furniture in kitchens and dining rooms or in men’s corners and studies. The object’s surface coating was thoroughly examined under daylight and UV light in order to identify the degree of any previous repairs or modifications. The conclusion of this examination classified the surface coating as showing no symptoms that would call into doubt the authenticity or coherence of this surface coating. The object thus met the criteria established by the project for inclusion in the research (determination of the object’s date and provenance and the intactness of the surface coating).

**Figure 6:**
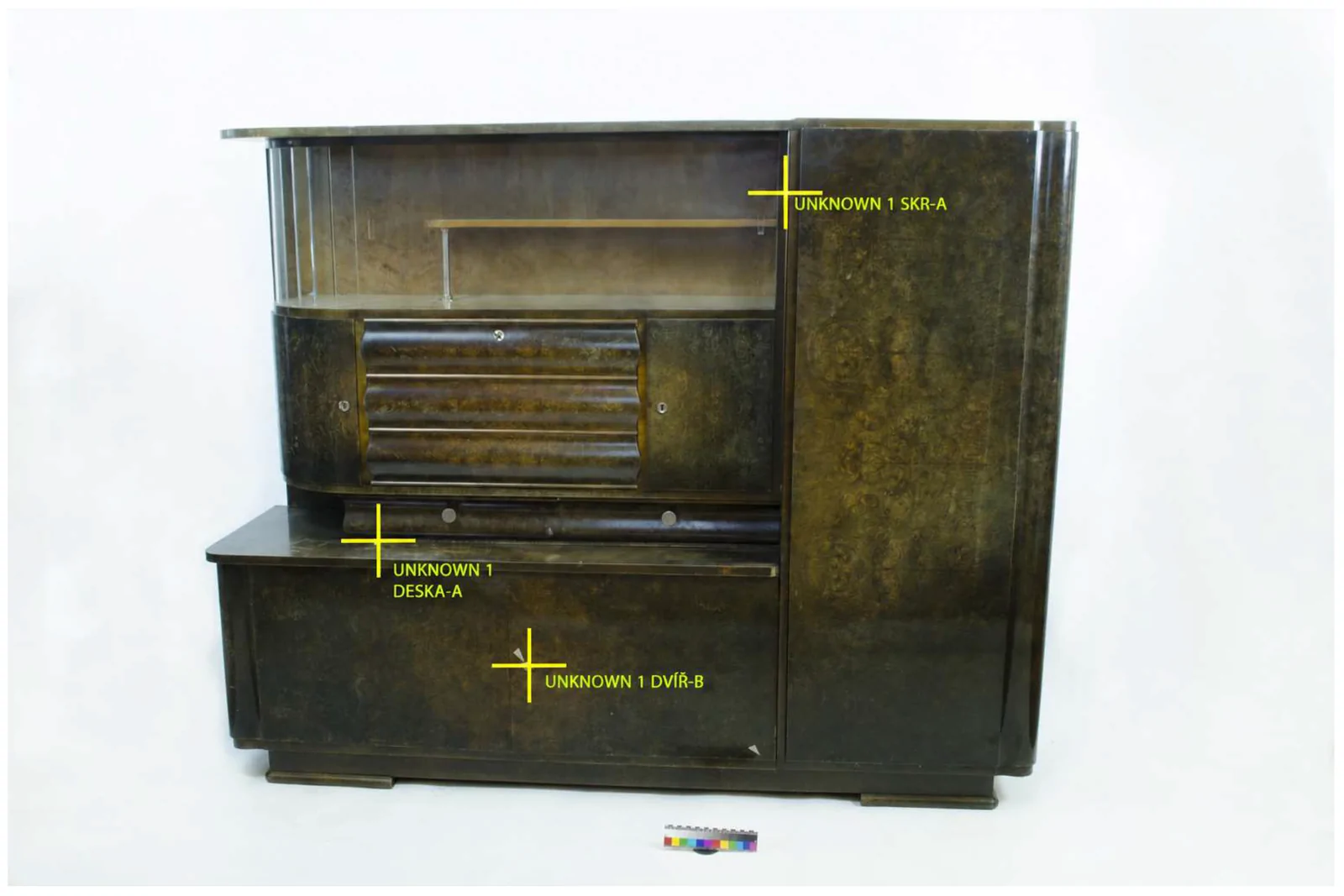
Historical Czech veneered storage furniture dated to approximately 1939 with marked sampling locations. The samples included in the present study were SKR-A, DVIR-B, and DESKA-A.

A total of seven surface coating samples were taken from the object, and only the three most relevant ones were included in this study. That is, those whose sampling location guarantees a high probability of authenticity without contamination by commercial cosmetics used for surface maintenance and renovation. The samples labeled DESKA-A and SKR-A were taken from a storage surface in a location that is normally covered by the cabinet body (construction) of the piece. The DVÍŘ-B sample was taken from the surface finish of the storage compartment door in a location that is normally covered by the keyhole plate. The historical furniture object and the sampling locations of the three samples included in this study are shown in Figure 6.

### 4.2. Sample Preparation

HPLC grade acetone and ethanol was purchased from Sigma-Aldrich (Prague, Czech Republic). Light decolorized, wax-free shellac (Kremer Pigmente GmbH & Co. KG, Aichstetten, Germany) and transparent nitrocellulose varnish (Lazurol Pragolesk C1037, Prague, Czech Republic) were used as model materials. The shellac component was prepared according to the archival shellac concentrate formulation corresponding to 2 kg of shellac per 4 L of ethanol. Model shellac/nitrocellulose mixtures were then prepared at mass ratios of 25:75, 50:50, and 75:25 (*w*/*w*) and dissolved in ethanol/acetone (50:50, *v*/*v*). The prepared mixtures were then kept under ambient office conditions for approximately two years before analysis to simulate more realistic ageing of mixed historical coating systems. This interval was not a controlled accelerated-ageing treatment; it allowed early naturally occurring changes before measurement but cannot reproduce the extent or heterogeneity of ageing in historical coatings.

The prepared solutions were analysed by ATR-FTIR without any further sample treatment. For ASAP-MS analysis, however, 50 µL aliquots of all solutions (pure varnishes and model mixtures) were further diluted with acetone to a final volume of 1000 µL and filtered through 0.22 µm PTFE syringe filters. Blank measurements were performed using capillaries dipped in acetone. Real varnish samples (~1 mg) were extracted with 500 µL of ethanol/acetone (50:50, *v*/*v*), sonicated for 15 min, centrifuged at 14,000 rpm for 1 min, and filtered through 0.22 µm PTFE syringe filters. Subsequently, 200 µL of each extract was transferred to a new vial and diluted with acetone to a final volume of 1000 µL prior to ASAP-MS analysis.

### 4.3. Instrumentation

#### 4.3.1. Fourier Transform Infrared Spectroscopy

Samples were analysed by Fourier transform infrared spectroscopy in attenuated total reflectance mode using a Nicolet iS50 FTIR spectrometer (Thermo Fisher Scientific, Waltham, MA, USA). Spectra were acquired over the range of 400–4000 cm^−1^. Each measurement lasted 30 s, with an acquisition rate of 1 scan s^−1^. Data were acquired using OMNIC 9 software (Thermo Fisher Scientific, Waltham, MA, USA) and exported to Excel to construct a sample-by-wavenumber matrix. The complete recorded spectral range was used for principal component analysis (PCA), without manual selection of diagnostic bands. Before PCA, the matrix was mean-centred and scaled to unit variance using the StandardScaler implementation in scikit-learn. PCA was subsequently performed in Python v3.12. 

#### 4.3.2. Atmospheric Solid Probe Analysis Mass Spectrometry

Atmospheric solid probe analysis (ASAP) was performed in a negative mode on a SELECT SERIES Cyclic IMS QTOF (Waters Corp., Wilmslow, UK). Sodium iodide 2 µg/mL in propan-2-ol/water (50/50, *v*/*v*) was used for the mass calibration (*m*/*z* 50–2000). For sample introduction, a glass capillary (length 100 mm, diameter 2.0 mm) was briefly dipped into the prepared solution and inserted into the ASAP probe assembly. To enhance low intensity signals of volatile compounds, a temperature program was applied: the default desorption temperature was 200 °C and was increased to 500 °C after 10 s. Data acquisition time was 1 min. The instrumental parameters were set as follows: corona voltage 2.00 kV, corona current 2.0 µA, cone voltage 30 V, source temperature 100 °C, desolvation temperature 200–500 °C, cone gas flow 30 L/h, desolvation gas flow 500 L/h, trap CE 6 V, transfer CE 4 V, stepwave body gradient 20 V, ion guide TW pulse height 0.4 V, trap TW pulse height 1 V, trap entrance 2 V, trap bias 2 V, trap DC −4 V, post trap gradient 7 V, post trap bias 35 V, stepwave RF 200 V, ion guide RF 300 V, driftcell RF 300 V, transfer RF 200 V. Data were acquired and processed using MassLynx v4.2 (Software Change Note 1016, Waters Corp., Wilmslow, UK). After export from MassLynx to Microsoft Excel as *m*/*z*–intensity tables, the resulting matrix was processed in Python using PCA and PLS-R, with data pretreatment described in detail in Section 4.4.

### 4.4. ASAP-MS Data Processing

#### 4.4.1. Data Processing

Model samples of the pure varnishes and their binary mixtures were analysed in eight replicates in random order. Real samples were analysed in duplicate as an internal quality-control measure. Raw mass spectra were exported from MassLynx as *m*/*z*–intensity tables and converted into a sample × feature matrix by binning the *m*/*z* axis onto a fixed grid with a bin width of 0.02 Da. Intensities falling within each bin were summed, and bins not observed in a given spectrum were filled with zeroes.

Background signals were removed using a blank-based filtering procedure. A global blank threshold was defined as the 99.5th percentile of all non-zero intensities observed across blank replicates. A given *m*/*z* bin was retained only if its intensity exceeded this threshold in at least two training replicates belonging to the shellac, nitrocellulose, or model-mixture groups; otherwise, the bin was discarded. After blank filtering, each spectrum was normalized by total ion current to compensate for differences in overall signal intensity between measurements. The normalized data were then transformed using the decimal logarithm. To avoid undefined values at zero intensity, a constant offset ε was added before transformation; ε was defined as one-tenth of the minimum non-zero total-ion-current-normalized intensity in the processed matrix. TIC normalization was used to compensate for differences in total ion signal between spectra and thus improve comparability across samples. Logarithm transformation was then applied to compress the dynamic range and reduce the influence of highly abundant ions. These processing steps were applied to improve comparability between spectra and to stabilize variance prior to multivariate analysis.

#### 4.4.2. Model Verification

Prior to PLS-R modelling, the pretreated data were subjected to principal component analysis (PCA) as an exploratory tool to assess replicate consistency, sample grouping, and potential outliers. PCA was fitted on the training set (shellac, nitrocellulose, and their mixtures) and subsequently applied to all samples including blanks.

Semi-quantitative modelling was performed using partial least squares regression (PLS-R), in which the response variable represented the shellac fraction in the model samples. Nitrocellulose was assigned a value of 0.00, pure shellac a value of 1.00, and the binary mixtures intermediate values of 0.25, 0.50, and 0.75 according to their composition. To reduce dimensionality and limit overfitting, only the 300 most variable *m*/*z* bins within the training dataset were retained for modelling. To avoid data leakage, this variance-based feature selection was nested within the leave-one-out cross-validation (LOOCV) procedure: in each validation cycle, the variables were ranked using only the (*n* − 1) calibration samples, the PLS-R model was fitted on this fold-specific subset, and the omitted sample was predicted using the same variables.

Model performance was evaluated from the LOOCV predictions using the root mean square error of cross-validation (RMSECV), the mean absolute error of cross-validation (MAECV), the cross-validated coefficient of determination (Q^2^), and the coefficient of determination calculated from the cross-validated predictions (R^2^CV). The linearity and potential systematic bias of the predictions were further assessed from the slope and intercept of the regression between the reference and LOOCV-predicted values. In addition, two auxiliary indicators were used to characterize overall signal quality in the processed dataset: the median relative standard deviation of TIC-normalized feature intensities within the shellac replicate set, and the mean blank-to-shellac total ion current ratio.

The effect of model complexity was examined across one to eight latent variables. The final number of latent variables was selected from the Q^2^ and RMSECV profiles as the lowest value beyond which further model expansion provided no substantial improvement in predictive performance. Based on this criterion, four latent variables were selected for the final model as a parsimonious compromise between model robustness and predictive accuracy.

To assess whether the observed predictive performance could arise by chance, model validity was additionally examined by permutation testing (n = 500). In each permutation, the response vector was randomly shuffled while the predictor matrix remained unchanged, and Q^2^ was recalculated using the same LOOCV-based modelling procedure with four latent variables and 300 selected features. The significance of the final model was then evaluated by comparing the observed Q^2^ with the distribution obtained from the permuted models.

After validation, the final PLS-R model was fitted to the complete training dataset and used to project the real varnish samples as external unknowns. These samples were preprocessed identically to the model samples, using the blank threshold, retained feature set, and transformation constant derived from the training data. For each object analysed in duplicate, the mean predicted shellac mass fraction and the corresponding standard deviation were reported.

## 5. Conclusions

The developed ASAP-MS workflow combined with PLS-R enables reliable semiquantitative estimation of contents of shellac/nitrocellulose in mixtures. The model showed high predictive performance and low systematic bias, confirming that non-targeted ASAP-MS fingerprints contain sufficient information for semiquantitative interpretation when supported by appropriate data preprocessing and chemometric modelling.

ATR-FTIR/PCA was included as an established qualitative reference approach in conservation practice, placing the proposed ASAP-MS workflow in the context of established analytical approaches. Whereas ATR-FTIR/PCA provided qualitative discrimination, ASAP-MS/PLS-R independently enabled semiquantitative estimation of shellac/nitrocellulose ratios. The comparison illustrates the additional compositional information offered by the proposed workflow. The workflow was also successfully applied to real microsamples from an authentic historical furniture object. The obtained estimates of the relative shellac/nitrocellulose composition provided practically relevant information for conservation practice, because knowledge of the varnish system can support the selection of appropriate restoration treatments and reduce the risk of chemically unsuitable interventions.

The method is inherently limited by its semiquantitative character, the use of simplified binary calibration systems, and variability associated with ASAP ionization and sample introduction. An additional constraint arises from the use of naturally aged model samples, where the chemical composition is not strictly controlled and may evolve over time, potentially affecting model calibration and transferability.

Despite these limitations, the approach proved to be well-suited for the studied type of varnish systems, demonstrating robust performance on both model and real samples. It therefore represents a practical and analytically meaningful strategy for estimating binder composition in historically relevant, non-ideal samples typical of cultural heritage materials.

## Figures and Tables

**Table 1:** PLS-R predicted shellac mass fractions in historical varnish samples. Values represent the mean and standard deviation (SD) of replicate measurements (n = 2).

Sample	Mean Shellac	SD Shellac
SKR-A	0.7807	0.0038
DVIR-B	0.6392	0.0370
DESKA A	0.3959	0.0017

For DVIR-B, both approaches indicate shellac dominance. The discrepancies observed for SKR-A and DESKA A—where the FTIR PCA class proximity and the PLS-R numerical estimates do not straightforwardly agree—cannot be resolved on the basis of the present data, but they further underscore the limitation of qualitative PCA assignment when samples occupy ambiguous score-plot positions.

## Data Availability

Raw FTIR spectra, merged ASAP-MS spectra and complete Python code are available in the Zenodo repository at https://doi.org/10.5281/zenodo.20708500 (accessed on 15 June 2026) under a CC BY 4.0 license.
